# Cell death and inflammation during obesity: “Know my methods, WAT(son)”

**DOI:** 10.1038/s41418-022-01062-4

**Published:** 2022-09-29

**Authors:** Ximena Hildebrandt, Mohamed Ibrahim, Nieves Peltzer

**Affiliations:** University of Cologne, Faculty of Medicine, Centre for Molecular Medicine Cologne (CMMC); Department of Translational Genomics and; Cologne Excellence Cluster on Cellular Stress Responses in Aging-Associated Diseases (CECAD), Cologne, Germany

**Keywords:** Ageing, Cell death and immune response, Chronic inflammation

## Abstract

Obesity is a state of low-grade chronic inflammation that causes multiple metabolic diseases. During obesity, signalling via cytokines of the TNF family mediate cell death and inflammation within the adipose tissue, eventually resulting in lipid spill-over, glucotoxicity and insulin resistance. These events ultimately lead to ectopic lipid deposition, glucose intolerance and other metabolic complications with life-threatening consequences. Here we review the literature on how inflammatory responses affect metabolic processes such as energy homeostasis and insulin signalling. This review mainly focuses on the role of cell death in the adipose tissue as a key player in metabolic inflammation.

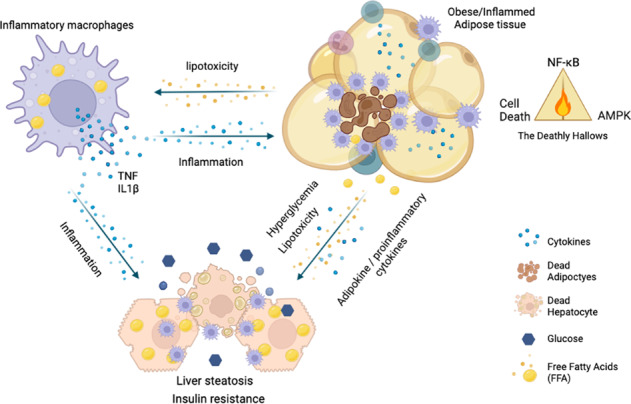

## Facts

- Obesity induces cell death and inflammation in the adipose tissue.
- Cell death within the adipose tissue induces inflammation-associated metabolic syndromes.
- Cell death machinery and NF-κB-mediated inflammation regulate energy homeostasis and insulin sensitivity.
- The crosstalk between adipocytes and adipose-tissue macrophages initiates systemic metabolic inflammation during obesity.

## Open questions

- Is cell death required to induce metabolic inflammation?
- Is there a “healthy” and an “unhealthy” way to die during excessive lipid uptake? And does it depend on the metabolic organ in which these events happen?
- Does activation of cell death machinery directly modulate metabolic processes, such as energy expenditure, during obesity?

## Introduction

Obesity is now considered a global disease as it affects over 1.9 billion people worldwide [1]. Obesity is a state of low-grade chronic inflammation that causes an array of different metabolic disorders, including insulin resistance (IR), Type 2 Diabetes, hypertension, cardiovascular disease, dyslipidemia and even cancer [2]. In recent years, studies have demonstrated a strong link between overnutrition and activation of the innate immune system as the leading cause of energy imbalance in most organs [1].

The white adipose tissue (WAT) is an endocrine and lipid storage organ that plays a pivotal role in obesity-associated disorders. Efficient lipid storage prevents ectopic lipid deposition and toxic lipid accumulation (lipotoxicity) in non-specialised organs, such as muscle, liver and heart, and it correlates with preserved metabolic function [3]. The WAT is mainly composed of preadipocytes or adipocyte precursors (AP) and adipocytes as well as of different types of immune cells, including macrophages, dendritic cells, T cells and B cells. Immune cells in WAT collectively monitor and maintain adipocyte integrity, metabolic function and hormonal sensitivity [4, 5]. Macrophages are the most abundant innate immune cells infiltrating and accumulating into WAT; they constitute up to 40–50% of all WAT cells. During obesity, adipose tissue macrophages (ATM) are polarized into pro-inflammatory M1-like macrophages and secrete many pro-inflammatory cytokines, such as TNF, capable of impairing insulin signalling, therefore, promoting the progression of IR. Although many factors are involved in the increased recruitment of macrophages into WAT during obesity, it is mainly attributed to adipocyte death. Macrophages are generally found surrounding dead adipocytes forming the typical crown-like structure (CLS), and the presence of these structures is directly associated with IR in mice and men [6, 7].

**Fig. 1:**
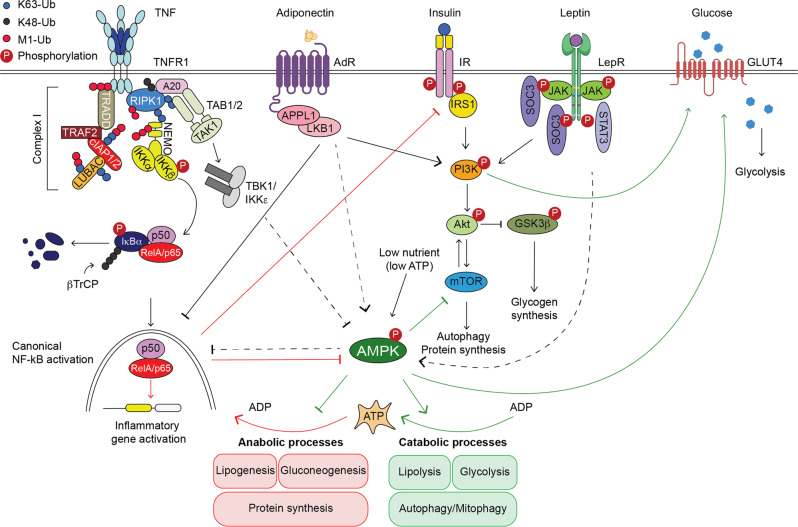
Crosstalk between NF-κB and metabolic pathways. TNF induces NF-κB activation via the assembly of a complex which includes RIPK1, the E3 ligases cIAP1/2 and LUBAC which ubiquitinate and stabilise the complex, and the kinase complexes IKKɑ/β/ɣ (NEMO) and TAK1/TAB1/2. The thereby activated IKKɑ/β/ɣ(NEMO) complex promotes degradation of the inhibitor of kB (IκB) and activation of NF-κB (here exemplified as p50/RelA/p65) which translocates to the nucleus and activates target genes. Activation of NF-κB then results in the inhibition of AMPK and Insulin signalling (red arrows and blockers). AMPK is a sensor of low ATP and induces a plethora of catabolic processes to uptake/produce energy whilst blocking processes that required energy (ATP) (green arrows and blockers). Activation of both Adiponectin Receptor (AdR) and Leptin Receptor (LepR) induces AMPK phosporylation and glucose uptake. Insulin sensing by Insulin Receptor (IR) is crucial for glucose uptake by activating Akt and allowing the activity of glucose transporters (GLUT4).

Adipocytes do not only play a role in lipid storage, but also on metabolism and inflammation through the secretion of cytokines and adipokines, such as leptin and adiponectin [8]. Leptin is considered to be the satiety hormone and it has pro-inflammatory functions [9, 10]. Adiponectin, in contrast, has anti-inflammatory properties by downregulating cytokines, such as TNF, MCP-1, and IL-6 [11, 12]. In obese individuals leptin plasma levels raises while adiponectin tends to decrease [13, 14]. Adipokines regulate energy expenditure as well as glucose and lipid metabolism through the metabolic regulator, AMP-activated protein kinase (AMPK) [15]. AMPK is an intercellular energy sensor, which is sensitive to AMP:ATP ratios [16]. AMPK promotes energy conservation by shutting down anabolism (gluconeogenesis, fatty acid synthesis) and activating catabolic pathways (β-oxidation, ATP production) [17] (Fig. 1). Even though this kinase responds to several stimuli that exhaust ATP levels in cells, it can also be phosphorylated, and activated, in response to adiponectin and leptin stimulation in organs such as skeletal muscle [18, 19]. The only exception to this, is the hypothalamus, where leptin acts by decreasing AMPK activity, potentially explaining why leptin specialises in suppressing food intake [19].

In obesity, there is a dynamic remodelling of the WAT in which adipocytes can either increase in size (hypertrophy) or in number, following differentiation from AP, or adipogenesis, (hyperplasia). WAT can be classified in two main compartments, subcutaneous (SAT) and visceral (VAT). Each of them bearing specific metabolic functions and characteristics. They present different patterns of gene expression, including genes involved in adipocyte function and development [20]. Notably, VAT-APs are more resistant to differentiation into adipocytes and are more sensitive to cell death than SAT-APs [21]. This phenomena can also greatly contribute to inflammation and metabolic syndromes [8]. In general terms, whereas SAT expands by adipocyte hyperplasia, VAT predominantly expands by adipocyte hypertrophy in response to excess food intake [22]. Indeed, VAT is the fat depot that undergoes major cell death and inflammatory procceses [23]. Hypertrophic adipocytes secrete inflammatory cytokines such as TNF and IL-6, causes recruitment and activation of immune cells whilst reducing adiponectin and anti-inflammatory cytokines production [24, 25]. This state of low-grade chronic inflammation eventually results in lipotoxicity, systemic inflammation and metabolic syndromes. Furthermore, activated macrophages during obesity, although at first essential for healthy tissue expansion and remodelling, when sustained they can lead to fibrosis and impaired adipogenesis [21]. These events result in a vicious cycle of inflammation, cell death and metabolic dysbalance that together cause metabolic syndromes. Notably, this condition also promotes a protumorigenic microenvironment that induces or supports tumour growth in cancers that are linked to obesity such as breast, liver and colon carcinomas [26].

Inflammation, cell death and metabolic processes are highly interlinked processes during obesity and a tight balance between these processes is crucial to prevent metabolic diseases. Here we review the literature of the signalling events governed by the pleiotropic immune mediator, TNF, and a pathogen sensing system, the inflammasome, during obesity with a focus on the current knowledge regarding cell death regulation in the WAT and its impact on metabolic inflammation.

## The horror at their crimes is lost in the admiration at their skills: culprits TNF and IL-1β

The key importance of TNF signalling in obesity-induced inflammation and metabolic complications was vastly demonstrated in animal models and also in humans. Indeed, although still a matter of debate, TNF neutralisation improves glucose homeostasis and reduces diabetes risk in human patients [27]. Likewise, IL-1β is an important regulator of inflammation during obesity as its neutralisation ameliorates obesity-induced inflammation [28].

**Fig. 2:**
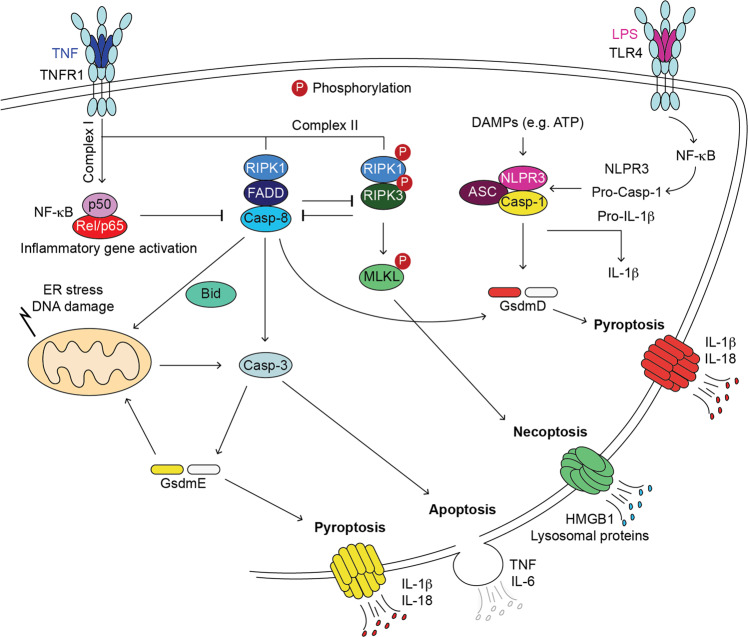
Different ways to die upon activation of death receptors of the TNFR1 family. Under pathological conditions, TNF can induce the formation of cell death complexes. Apoptosis is induced by formation of a RIPK1/FADD/Caspase-8 (Casp-8) complex. This results in cleavage and activation Casp-3 and apoptosis or, in certain conditions, Gasdermin D (GsmdD) and pyroptosis. Necroptosis is induced by recruitment of RIPK1 and RIPK3 which are activated by autophosphorylation. This leads to phosphorylation of MLKL which subsequently forms pores in the membrane. Canonical pyroptosis requires a priming event to upregulate the expression of inflammasome components, NRLP3, ASC and Caspase-1, such as TLR4 activation by Lipopolysaccharides (LPS). Pyroptosis is induced upon activation of NLPR3 by DAMPs such as ATP. Inflammasome formation consists of oligomerised NRLP3/ASC/pro-Caspase-1 (Casp-1). This platform leads to activation of Casp-1 which cleaves GsdmD that forms pores in the membrane and induces pyroptosis. Casp-1 also processes pro-IL-1β maturation which is then released by GsdmD pores. Pyroptosis can also be induced by a GsdmE, which can be cleaved and activated by Casp-3. GsdmE can amplify cell death responses by forming pores not only in cellular membranes but also in the mitochondrial membrane. The cell death programs, other than requiring the expression and activation of different proteins, are characterised by their specific secretomes. P: Phosphorylated protein. NF-κB activation prevents cell death complex formation. Apoptotic and Necroptotic machineries negatively regulate each other.

The signalling cascade that is unleashed following binding of TNF to its cognate receptor, TNFR1, results in activation of the nuclear factor κB (NF-κB) and mitogen-activated protein kinase (MAPK) via the formation of a receptor signalling complex, also known as complex I [29] (Figs. 1 and 2). This complex facilitates the activation of IKKɑ/β/ɣ(NEMO) and TAK1/TAB1/2 complexes. This leads to the transcriptional activation of genes, amongst which chemokines and cytokines including TNF itself, IL-6, and other prosurvival proteins, such as BCL2 and cFLIP. In principle, the latter prevents induction of cell death via intrinsic or extrinsic pathways, respectively [30].

TNF, in certain circumstances, can also induce cell death by recruitment of Caspase-8 and RIPK1 or TRADD to FADD to form the so called, complex II or cell death complex [31] (Fig. 2). This platform induces apoptosis via the activation of the effector caspase, Caspase-3 [32]. Active Caspase-8 can also crosstalk with the mitochondrial cell death pathway via the cleavage of Bid which can induce mitochondrial permeabilization [33]. Apoptosis is not the only cell death modality that can be induced by death receptors. RIPK1 can further recruit RIPK3 which leads to activation of the pseudokinase MLKL that oligomerises to form pores in the membrane ultimately leading to necroptosis [34]. Necroptosis is a highly inflammatory type of cell death [35, 36] and, importantly, it results in a secretome that is different from the secretome of apoptotic cells [37]. Notably, necroptosis is inhibited by Caspase-8 via the cleavage-mediated inactivation of RIPK1. Thus, loss of Caspase-8 unleashes necroptosis in the developing embryo resulting in embryonic lethality [38, 39].

Another type of cell death, that was best described to occur in immune cells, can be triggered as a result of Caspase-8 and -3 activation. Emerging evidence shows that proteins called Gasdermins are substrates of these caspases [40–43]. Gasdermin family members, which include Gasdermin A, B, C, D and DFNA5/Gasdermin E, are pore-forming proteins that allow the release of mature IL-1β and induce a type of cell death called pyroptosis upon cleavage-mediated activation [44]. Under certain pathological conditions, Caspase-8 can directly cleave and activate Gasdermin D or indirectly, via activation of Caspase-3, Gasdermin E. Gasdermin E is currently described as a bona-fide Caspase-3 substrate that mediates secondary necrosis/pyroptosis downstream of mitochondrial cell death activation [40–43]. This may in part explain the inflammatory character of mitochondrial cell death activation which was long thought to be immunologically silent. Notwithstanding, the main source of mature IL-1β is produced and secreted by the canonical inflammasome activation following a priming signal, such as activation of Toll-like receptor (TLR4) by lipopolysaccharides (LPS) derived from bacteria leaking from gut or by free fatty acids (FFA) in the context of obesity. This priming event is required to induce the expression of NF-κB-driven NLRP3, pro-Caspase-1 and pro-IL1β [45]. NLRP3 together with ASC and pro-Caspase-1 assemble in a platform called cannonical inflammasome following NLRP3 activation by damage associated molecular patterns (DAMPs) such as ATP. There are, however, other sensors that can form a variety of inflammasome platforms. Whereas the NLPR3 senses DAMPs, the NLRP1 sense viral particles and dsRNA, NLRC4 senses pathogen associated molecular patterns (PAMPs) and AIM2 senses dsDNA [46]. Inflammasome formation results in the activation of Caspase-1 that cleaves pro-IL1β and Gasdermin D. Activated Gasdermin D oligomerises in the membrane and forms pores from which mature IL1β, among other cytokines, is released (Fig. 2) [45].

## It is a mistake to confound mystery with misunderstanding: the NF-κB conundrum

Obesity is associated with inflammatory changes in WAT that lead to IR, which is the inability to sense insulin and uptake blood glucose and other nutrients. This is the primary cause of several metabolic syndromes such as Type 2 Diabetes [8]. During obesity, the NF-κB signalling pathway is activated in metabolic tissues and has important implications in obesity-induced IR [47–49]. In response to overnutrition, NF-κB signalling does not only contribute to systemic inflammation by promoting the generation of inflammatory cytokines and chemokines, but also to insulin resistance by directly interfering with the insulin signalling pathway [47] (Fig. 1). For example, IKKβ directly phosphorylates and inhibits the insulin receptor substrate (IRS), an essential adaptor of the activated insulin receptor (IR) (Fig. 1) [47]. The latter resulting in a weak or null response to insulin.

Our understanding of how inflammatory signalling is affected in different metabolic tissues derives from animal models and grew exponentially with the availability of conditional transgenic mice (Table 1). A pioneer study found that reduced IKKβ signalling, either by salicylate inhibition or decreased IKKβ expression (*Ikkβ*^*+/−*^), is accompanied by decreased FFA and improved insulin sensitivity in genetically obese *db/db* mice and *Zucker*^*fa/fa*^ rats [50]. Salicylate treatment was able to overcome the inhibition of insulin signalling by TNF stimulation, implying that the IKKβ pathway may contribute to IR in Type 2 Diabetes and obesity by impinging on insulin signalling [50]. Another seminal study showed that deficiency of IKKβ in hepatocytes improves glucose tolerance and insulin sensitivity upon high fat diet (HFD). The authors show that IKKβ negatively regulates insulin signalling in hepatocytes since IKK deficiency in these cells led to an improved insulin signalling in mice under HFD or in genetically obese *ob/ob* mice [51]. IKKβ deficiency in hepatocytes did not affect insulin sensitivity in other metabolic organs such as muscle and WAT. Likewise, mice bearing loss of IKKβ in myeloid cells retain global insulin sensitivity and were protected from IR during HFD or in *ob/ob* mice [51]. This implies that NF-κB signalling in myeloid cells plays a crucial role in systemic obesity-induced inflammation, whereas the role of NF-κB signalling in the liver is restricted to this tissue. In line with this, inhibition of NF-κB by siRNA against p65 subunit protects HFD-fed mice from hepatic steatosis and insulin resistance without affecting body weight gain. Notably, p65 siRNA predominantly affected NF-κB transcriptional activity in the liver but, importantly, not in the WAT [52]. Interestingly, the authors highlighted a crosstalk between NF-κB signalling pathway and liver AMPK/mTOR/autophagy pathway since p65 siRNA enhanced the activity of AMPK and activated genes involved in autophagy such as *beclin1* in the liver (Fig. 1). This finding links NF-κB with metabolic pathways and energy homeostasis [52]. A different report suggests that expression of NF-κB in ATM prevented ATM death by induction of anti-apoptotic gene expression during obesity [53]. The latter is supported by the recent report showing that A20 deficiency in myeloid cells results in increased NF-κB activation and, although this was associated with increased inflammation, the typical CLS surrounding dead adipocytes were absent upon HFD [54]. Notably, loss of A20 in myeloid cells results in a protection against HFD-induced IR and increased energy expenditure due to elevated ATM metabolism [54] (Table 1).

**Table 1:** Role of NF-κB activation in obesity-induced inflammation.

Tissue	Target	Model	Major Findings	Reference
	Protein			
Full body	NF-κB	HFD or *Lep*^*ob/ob*^	↑Antiapoptotic proteins in WAT	[53]
			↑NF-κB in ATMs	
			↓cleaved Casp3 in WAT and ATMs cell death.	
		p65 inhibition by siRNA in HFD	↑pAMPK in liver but not in WAT.	[52]
			= Body weight gain.	
			↓NF-κB and mTOR.	
			Ø Hepatic steatosis and insulin resistance.	
		*p50*^−/−^ in HFD	↑NF-κB, inflammation, ATMs, food intake and energy expenditure.	[57]
			↓Body weight and differentiation capacity.	
			↓Leptin and PPARγ in WAT.	
	IKKβ	IKK-β inhibition	*Zucker*^*fa/fa*^ rats and *Lep*^*ob/ob*^ mice:	[50, 99]
			↑Glucose tolerance.	
			= Body weight and food intake.	
			↓Insulin resistance, Normal and fasting glucose, FFA, triglycerides [50]	
			HFD:	
			↓Insulin resistance [99]	
		*Ikk-β*^*+/−*^ in HFD or *Lep*^*ob/ob*^ mice	Ø insulin resistance [50]	
	IKKε	HFD	↑IKKε expression in WAT, liver, and macrophages.	[55]
			*Ikkε*^**−/−**^**:**	
			↓WAT inflammation, hepatic steatosis and insulin resistance.	
	TBK1 / IKKε	HFD	↑IKKε and TBK1 in WAT and liver during obesity.	[56]
			TBK1/IKKε inhibition:	
			↑energy expenditure, thermogenesis, and insulin sensitivity.	
			↓Body weight and hepatic steatosis.	
Adipose tissue	NF-κB	*p65*^OE^ in HFD	Lean: ↑NF-κB, inflammation, ATMs and energy expenditure.	[57]
			= food intake and insulin sensitivity.	
			↓Body weight.	
			Obese: ↑NF-κB, inflammation, ATMs food intake, energy expenditure and insulin response.	
			↓Body weight.	
	IKKβ	Over expression of Ikkβ in adipose tissue in HFD	Lean: ↑glucose tolerance, insulin tolerance, and energy expenditure.	[58]
			↓Blood glucose levels.	
			Obese: ↑systemic and tissue inflammation.	
			↓Weight and triglycerides of WAT	
			↓Triglycerides in liver and muscle.	
		HFD	↑IL-13 in WAT [60]	[59, 60]
			*Ikkβ*^*A-KO*^ :	
			↑WAT inflammation, ATMs infiltration glucose intolerance and insulin-resistance [59, 60]	
			↑lipolysis, FFA circulation, hepatic cholesterol and primary adipocytes cell death [59]	
			= Body weight, food intake and energy expenditure [59, 60]	
			↓Epididymal fat mass [59] and IL-13 (anti-inflammatory protective role in adipocytes) [60]	
	TBK1	HFD	↑TBK1 in adipocytes, lipid storage	[100]
			↓AMPK	
			*Tbk1*^*A-KO*^:	
			↑p-AMPK, pAKT, energy expenditure, WAT inflammation, ATMs infiltration, insulin resistance and glucose intolerance.	
			= Body weight.	
			↓Fat mass, adipocyte size.	
Liver	NEMO / IKKα/β	*Nemo*^*hep-KO*^	Spontaneous steatohepatitis, and tumorigenesis [62–64]	[62–64]
			HFD:	
			↑↑↑steatosis, inflammation, apoptosis and tumorigenesis in liver [62]	
			↓PPRƔ [62]	
			*Nemo*^*hep-KO*^*NF-κB*^*hep-KO*^ *or Nemo*^*hep-KO*^*Ikkα/β*^*hep-KO*^**:**	
			↑Apoptosis, spontaneous steatohepatitis and hepatocarcinoma [63, 64]	
			NEMO deletion rescues IKKα/*β*^*hep-KO*^ from cholestasis by Ø liver necrotic foci (↓RIPK3 ↑cleaved Caspase-3 levels) but mice develop hepatocarcinoma) [64]	
			*Nemo*^*hep-KO*^*Ikk*β^*ca-hep*^**:**	
			↑IKK-β /NF-κB Ø liver damage, hepatocarcinogenesis and hepatocyte apoptosis by ØRIPK1 activity.	
			NEMO’s protective role is partially dependent of NF-κB [63]	
	IKKβ	HFD	*Ikk-β*^*hep-KO*^	[51, 99]
			↑Insulin resistance in muscle and WAT [51]	
			= Liver insulin response [51]	
			*Ikkβ*^*hep-OE*^ **:**	
			↑NF-κB, proinflammatory cytokines (IL-6, IL-1β and TNF-α), lipid accumulation and insulin resistance [99]	
			*Ikkβ*^*hep-OE*^*IκBα*^*SR*^**:**	
			↓IκBα ↓Inflammation on *Ikkβ*^hep-OE^ and WT [99]	
Myeloid cells	IKKβ	*Ikk-β*^*mye-KO*^ in HFD	= Global insulin sensitivity.	[51]
			Ø Insulin resistance.	
	MVP (NF-κB inhibitor)	HFD	↑MVP in ATMs.	[101]
			*Mvp*^*mye-KO*^**:**	
			↑NF-κB in macrophages, Insulin resistance, hepatic steatosis, atherosclerosis, macrophages infiltration and activation.	
	A20/RIPK3	HFD	*A20*^*mye-KO*^**:**	[54]
			↑NF-κB, inflammation in WAT, palmitate oxidation, ATMs, food and oxygen consumption.	
			↓CLS, FFA, Triglycerides, Cholesterol, blood glucose, insulin, leptin.	
			ØGain weight, glucose intolerance, insulin resistance.	
			A20^mye-KO^*Ripk3*^−/−^**:** same phenotype as *A20*^*mye-KO*^	

↑ increase, promote; = equals, not modification; ↓ decrease; Ø blocks.

*A-OE* Adipocytes over expression, *KO* knock-out, *A-KO* adipocytes KO, *ATMs* adipose tissue macrophages, *ca-hep* constitutively active in hepatocytes, *CLS* Crown-like structures, *FFA* free fatty acids, *hep-OE* hepatocytes overexpression, *hep-KO* hepatocytes KO, *HFD* high fat diet, *HSD* high sucrose diet, *mye-KO* myeloid cells (macrophages) KO, *OE* over expression, *siRNA* small interfering RNA, *SR* super repressor.

Other studies support a crosstalk between NF-κB signalling and metabolic pathways (Fig. 1), and how its activation amplifies the pro-inflammatory state associated with obesity [47]. Increased NF-κB activation in ATMs during HFD, correlates with an increased expression of IKKε, a known NF-κB target gene. IKKε-deficient mice gain less weight upon HFD and are protected against insulin resistance. These mice have reduced inflammation in the liver and WAT with reduced expression of proinflammatory genes in adipocytes and ATM. IKKε-deficient mice also display increased thermogenesis and enhanced expression of Uncoupling proteins (UCP) 1 an important enzyme in oxidative phosphorylation and downstream target of AMPK. Hence, the reduced inflammation could be associated with an overall amelioration of the metabolic status of the animals which results in decreased inflammation [55]. In line with this, a small molecule inhibitor of TBK1/IKKε axis, amlexanox, showed increased thermogenesis accompanied by elevated energy expenditure. Likely as a consequence of this, it resulted in weight loss, improved insulin sensitivity and decreased steatosis in obese mice fed with HFD [56] (Table 1).

The impact of NF-κB activation on metabolic syndromes appears to be highly cell type and tissue specific [47]. Whereas NF-κB activation in liver and ATMs is associated with increased inflammation and hindered energy expenditure, in adipocytes this association is less clear. Two studies show that overexpression of p65 specifically in adipocytes [57] or the constitutive activation of IKKβ in adipocytes [58] both lead to increased energy expenditure in basal conditions. These mice display, in addition, increased insulin sensitivity and are protected from excess body weight gain upon HFD despite suffering from exaggerated local and systemic inflammation. In stark contrast, two studies show that targeted deletion of IKKβ in adipocytes, whilst it does not affect body weight, food intake, and energy expenditure, it results in an exaggerated diabetic phenotype with systemic inflammation when challenged with HFD [59, 60]. Mice lacking IKKβ in adipocytes present dystrophic adipose tissue (lipodystrophy) and ectopic lipid deposition upon HDF, unveiling a role of IKKβ in adipocyte survival and adipose tissue remodelling during obesity [59]. This may indicate that although increased NF-κB during obesity regulates energy homeostasis, the complete loss of IKKβ in adipocytes, may results in excessive cell death since it has important implications in cell death regulation independently of its function in NF-κB activation [61] (Fig. 2). Of note, this similar observation was made with NEMO deficiency in the liver [62–64].

The body of literature highlighted here demonstrates that NF-κB signalling not only regulates inflammation during obesity but it is directly linked to energy expenditure by regulation of AMPK and glucose uptake/proliferation by regulation of insulin signalling (Fig. 1). In addition, it remains clear that NF-κB signalling and/or the IKK kinase complex can regulate cell death, inflammation and other metabolic signalling events in a tissue specific manner, which merely depends on the nature and function of the tissue. In an expanding WAT, a certain amount of NF-κB-mediated inflammation is advantageous to prepare the tissue to uptake lipids on demand and prevent FFA spillage by inhibiting AMPK (anabolic profile). However, in the liver, the contrary needs to happen in order to increase energy expenditure (catabolic profile) and avoid ectopic lipid deposition. For instance, excessive inflammation by NF-κB impairs AMPK-mediated energy expenditure resulting in major lipid deposition, feeding the inflammatory loop and inducing IR.

In conclusion, it is clear that NF-κB plays a unique role in metabolic inflammation depending on the cell type/organ. Yet, it is still uncertain what is the threshold of NF-κB activity that is either required for WAT remodelling or detrimental for energy and metabolic homeostasis. Most importantly, to what extent and how NF-κB signalling regulates cell death processes during obesity is currently not entirely understood.

## Examination of a crime scene: apoptosis and/or Necroptosis in WAT

It is currently accepted that adipocyte death is a key initial event that contributes to macrophage infiltration into adipose tissue and IR associated with obesity in both mice and humans [65]. Indeed, 90% of macrophages infiltrating the adipose tissue of obese animals and humans are arranged around dead adipocytes, forming characteristic CLS [6, 7]. Surprisingly, even though adipocyte death is such a common feature in WAT upon obesity, little is known about the impact of different types of cell death, in particular those induced by ligands of the TNF family.

The Fat Apoptosis Through Targeted Activation of Caspase-8 (FAT-ATTAC) model demonstrated the impact of Caspase-8-mediated apoptosis in the WAT. Induced expression and activation by dimerisation of Caspase-8 in this model resulted in local inflammation and adipocyte death which was accompanied by marked glucose intolerance [66]. This damage was totally reversible and the WAT from animals in which forced Caspase-8 activation does not persist can fully recover. Yet, glucose intolerance is not fully reversible. This suggests that, although endogenous preadipocytes can successfully differentiate and regenerate the adipose tissue, a transient stress in the WAT can have long-lasting or even permanent effects on systemic inflammation and metabolism. Despite displaying elevated food intake, FAT-ATTAC mice fail to gain weight when crossed with *ob/ob* mice. This indicates that the adipocytes from these mice have an impaired ability to uptake lipids independently of leptin signalling or food intake, and that this failure causes lipotoxicity in peripheral organs. FAT-ATTAC mice presented increased ATM infiltration and, curiously, the majority of these ATMs were alternatively activated anti-inflammatory M2-like macrophage upon Caspase-8 activation [67]. Hence, whilst apoptosis of adipocytes is sufficient to initiate a large influx of macrophages into WAT, they are of anti-inflammatory character, at least upon forced activation of Caspase-8 [67] (Table 2).

**Table 2:** Cell death proteins are implicated in homeostatic tissue response to obesity.

Cell death program	Target Protein	Model	Major Findings	reference
Apoptosis/Necroptosis	FADD	HFD or *Lep*^*ob/ob*^mice	*Fadd*^*A-KO*^:	[68]
			↑Energy expenditure and fatty acid oxidation, food intake, mitochondrial content in WAT, insulin sensitivity and glucose tolerance.	
			↓FFA, inflammation, hepatic steatosis, WAT mass, weight gain, ATMs	
	Caspase-8	*Casp8*^*A-OE*^	↑Apoptosis of adipocytes [66, 67].	[66, 67]
			↑M2 macrophages in WAT, TNF and MCP-1 in WAT [67].	
			= Body weight [67].	
			↓WAT mass and adiponectin [67].	
			*Lep*^*ob/ob*^:	
			↑Apoptosis in adipocytes, energy expenditure, plasma levels of glucose and triglycerides, glucose intolerance, food intake, WAT macrophages infiltration and hepatic steatosis.	
			↓Body weight, leptin and insulin levels [66].	
	cFLIP	NASH (HFD) or *Lep*^*ob/ob*^ mice	↓cFLIP only in liver	[71]
			*cFLIP*^*hep-KO*^(HFD mice):	
			↑body and liver weight, hepatic steatosis, inflammation, glucose and fatty acid uptake, plus fatty acid synthesis.	
			= Food intake.	
			↓Fatty acid β-oxidation, glucose and insulin tolerance	
			*cFLIP*^*hep-OE*^ (HFD Monkeys):	
			↑Liver function and fatty acid metabolism.	
			= Body weight.	
			↓Hepatic lipid accumulation, fibrosis and inflammatory response	
	BID	HFD or HSD	↑Caspase-3 cleavage.	[65]
			*Bid*^−/−^:	
			↑Insulin sensitivity.	
			= Gain weight.	
			↓Caspase activation, adipocyte apoptosis, ATMs and hepatic steatosis [48].	
	Cyclophilin D	*CyclophilinD*^−/−^ in HFD	↑Perilipin.	[102]
			Ø Adipocyte cell death.	
			= Glucose tolerance, inflammation, ATMs, insulin resistance, weight gain.	
			Mitochondria permeability does not play a role in HFD-induce inflammation	
	TAK 1	*Tak1*^*A-KO*^ in HFD or *Lep*^*ob/ob*^ mice	↑Apoptotic adipocytes, M2-like ATMs in WAT, energy expenditure, glucose tolerance, food consumption.	[103]
			↓Adipocyte numbers, gain weight and WAT weight	
	RIPK1	HFD or *Lep*^*ob/ob*^	↑*Ripk1* expression in obese mice [78, 79] and human [78].	[78, 79]
			*Ripk1* siRNA in HDF:	
			↑IL-10, iNKT cells infiltration and insulin sensitivity.	
			↓Hepatic inflammation, fat mass, total body weight and ATM [78].	
			RIPK1 inhibition (Nec-1) in *Lep*^*ob/ob*^:	
			↑Increased glucose tolerance	
			↓Fasting blood glucose insulin resistance, fat deposition, hepatic triglycerides	
			= Body weight, food intake and inflammation [79].	
	RIPK3	HFD	↑RIPK3 in WAT and liver of obese humans and mice [72, 104–106]	[72, 104–106]
			*Ripk3*^−/−^:	
			↑Caspase-8-dependent adipocyte apoptosis and WAT, inflammation, glucose intolerance [72, 104]	
			↑Liver injury, lipid accumulation in liver and insulin resistance [104].	
			↓insulin signalling in WAT [72].	
			*Ripk3*^−/−^ CDAA diet:	
			↑Apoptosis in liver and WAT, body weight, hepatic lipogenesis, fat accumulation, insulin/glucose, levels, MRC complex activity.	
			↓Inflammation and fibrosis in liver [106].	
			*Ripk3*^−/−^*Caspase-8*^*hep-KO*^:	
			= glucose tolerance and insulin resistance [72].	
			*Ripk3*^−/−^*Caspase-8*^−/−^:	
			↑glucose tolerance and insulin sensitivity [72].	
	MLKL	WD or HFD, *Lep*^*ob/ob*^ *LepR*^*db/db*^	↑MLKL levels in the liver of obese models [79].	[79, 107]
			Palmitic acid ↑MLKL expression, phosphorylation, oligomerization independently of RIPK3 [107].	
			MLKL regulates insulin signalling and sensitivity [79].	
			*Mlkl*^−/−^:	
			↓Body weight, insulin resistance, glucose intolerance [79, 107].	
			= Liver inflammation and levels of cell death [79].	
			↓Inflammation and liver injury [107].	
Pyroptosis	NLRP1	ND/HFD mice or Obese human	= NLRP1 levels in WAT of obese and lean human [93]	[93, 95]
			*Nlrp1*^*−/−*^ :	
			Spontaneous phenotype ↓IL-18. ↑adipose tissue, glucose intolerance, insulin resistance and	
			leptin levels.	
			HFD aggravates obesity, metabolic Syndrome, and steatosis in *Nlrp1*^*−/−*^ Mice [95]	
	NLRP3	HFD mice or Obese human	↑NLRP3 in liver of obese patients [84] and in mice and human WAT [84, 85, 108]	[84–87, 89, 90, 108, 109]
			↑WAT hypoxia and inflammation-related factors regulates NLRP3 expression [84].	
			*Nlrp3*^*−/−*^ *or Nlrp3* silencing.:	
			↑Adipogenesis, insulin sensitivity.	
			↓Inflammation, IL-1β, IL18, blood glucose, insulin levels, fibrosis [84, 89, 110]	
		LPS treatment (no diet)	↑NLRP3, IL-1β	[91]
			↓Mitochondrial function, browning in WAT	
	Caspase-1	HFD, *Lep*^*ob/ob*^ or *LepR*^*db/db*^	↑Caspase-1 in WAT and liver of obese humans and mice [85, 86, 89, 108]	[85, 86, 89, 90, 108]
			*Casp1*^−/−^:	
			↑Body weight, adiposity, insulin sensitive, inflammation, CCL2, Leptin and lipid oxidation.	
			= Lipid profiles, glucose intolerance, energy expenditure and liver weight.	
			↓Adiponectin and IL18 [85, 86].	
			Ex-vivo caspase-1 inhibition in human WAT:	
			↓IL1-β and IL-18 release [108].	
			Caspase-1 inhibition in *Ldlr*^*-/-*^ (Leiden Mice):	
			↑Insulin sensitivity.	
			= Body weight.	
			↓Inflammation in WAT and liver, CLS and hepatic steatosis [90].	
	Caspase-1/Caspase-11	HFD	↑Body weight and hepatic steatosis	[87]
	GsdmD	LPS injection or HFD [104] *Gsdmd*^*−/−*^ in MCD diet [102]	↑GSDMD and fragment GSDMD-N in WAT and Liver of obese humans and mice [98, 111].	[98, 111]
			GSDMD inhibitor (Melatonin):	
			↓GSDMD expression, NLRP3 activation, IL-1β in WAT [111].	
			*Gsdmd*^−/−^:	
			↑Lipolytic genes	
			↓Hepatic steatosis and lipogenic genes [98].	
	IL-1β	HFD or obese human samples	↑IL-1β in WAT and Liver of obese humans and mice [84, 109]	[84, 109]
			*IL-1β*^*−/−*^:	
			↑WAT weight.	
			= Body weight and Liver weight.	
			↓Hepatic steatosis, insulin resistance and infiltration inflammatory macrophages [109].	
	IL-18	ND mice or Obese human	↑ IL-18 in obese humans.	[96]
			*IL-18*^−/−^ :	
			Similar to *Nlrp1*^*−/−*^ spontaneous phenotype ↑adipose tissue, glucose intolerance and insulin	
			resistance, leptin levels. [96]	

↑increase, promote; =equals, not modification; ↓decrease; Ø blocks; p-phosphorylated.

*A-OE* Adipocytes overexpression, *KO* knock-out, *A-KO* adipocytes KO, *CDAA* choline-deficient L-amino acid-defined, *CLS* crown like structures, *Hep-OE* hepatocytes overexpression, *Hep-KO* hepatocytes KO, *HFD* high fat diet, *HSD* high sucrose diet, *MCD* methionine-choline deficient diet, *MRC* mitochondrial respiratory chain, *NASH* Non-alcoholic steatohepatitis, *si* small interfering RNA, *WD* Western diet (FFC diet, high in fat, fructose, and cholesterol), *ND* normal diet.

The FAT-ATTAC model clearly shows that apoptosis causes metabolic syndromes. Yet it is a rather acute model. Other models, however, also support the role of WAT apoptosis in metabolic inflammation. Mice deficient for FADD specifically in adipocytes are protected from glucose intolerance and IR in WAT, liver and muscle upon HFD feeding or when in a *ob/ob* background [68]. In addition, these mice display reduced WAT inflammation and increased energy expenditure. The authors attribute this phenotype to a role of FADD in suppressing PPARɣ-mediated gene expression since a mutation in FADD that abolishes PPARɣ-inhibition increased mitochondria, energy expenditure, and lipolysis in WAT by increasing cAMP levels [68] (Table 2). This suggests that FADD may interfere with adipogenesis and healthy expansion of the WAT during obesity. It is puzzling that FADD deficiency does not unleash necroptosis in adipocytes as it does in many other cells [40, 69, 70]. Yet, the authors did not focus on cell death in this study. Instead, this work provides robust evidence of a crosstalk between apoptosis and energy homeostasis.

In line with apoptosis playing a key role in obesity-induced inflammation, an interesting report shows that genetic deletion of Bid, which links the death receptor- with the mitochondrial- cell death pathways, significantly reduces caspase activation and adipocyte apoptosis in response to HFD. Thereby, BID-deficient mice displayed decreased to null ATM infiltration and were protected against the development of IR and hepatic steatosis. Interestingly, this protection was independent of body weight gain [65]. In the liver, cFLIP-hepatocyte-specific deletion protected mice and primates from HFD-induced steohepatitis [71] (Table 2).

Another study reports that RIPK3-deficient mice fed with HFD become, surprisingly, glucose intolerant and highly insulin resistant [72]. This was independent of RIPK3-mediated signalling in ATMs. RIPK3-deficient mice presented massive liver damage and apoptosis in both WAT and liver. This prompted the authors to test whether excessive apoptosis in the liver was responsible for this unexpected phenotype. However, whereas full body Caspase-8 deficiency protects RIPK3-deficient mice from HFD-induced inflammation and glucose intolerance, Caspase-8 deficiency in hepatocytes does not. This suggests that adipocyte apoptosis, but not hepatocyte apoptosis, may be the triggering event of metabolic inflammation in the absence of RIPK3.

The fact that RIPK3 loss induces apoptosis and sensitises mice to inflammation upon HFD is puzzling. RIPK3 is able to regulate inflammatory signalling independently of its role in cell death [31, 73, 74]. It is thus, intriguing, to speculate that, in adipocytes, RIPK3 serves as an inflammatory modulator rather than a killer protein. Notably, RIPK3 was reported to have a role in mitochondrial biogenesis and fatty acid oxidation in tumour associated macrophages [75–77]. Hence, RIPK3 may play a role in obesity-induced inflammation by mechanisms that go beyond necroptosis, possibly by preventing apoptosis in WAT or by direct regulation of inflammation and energy homeostasis (Table 2).

Although, the evidence so far suggests that the necroptosis in WAT does not occur or does not induce detrimental inflammation, other recent studies might indicate that the opposite is true. Necroptosis hallmarks are extremely rare (to detect) in human pathologies. Yet, obese individuals with or without Type 2 Diabetes showed massive expression of RIPK3 which correlated with activation of necroptosis as shown by increased phosphorylated MLKL [72]. In addition, a study performed on humans and mice discovered that polymorphisms in RIPK1 found in obese patients, positively correlate with increased body-mass index (BMI) [78]. These obesity-associated polymorphisms in the RIPK1 gene functionally result in elevated RIPK1 expression in adipose tissue in humans, and elevated RIPK1 expression in mice. Indeed, attenuation of RIPK1 expression using shRNA prevented body weight gain upon HFD and the metabolic dysfunctions associated with it, including inflammation and hepatic steatosis. In line with this, another study shows that inhibition of the kinase activity of RIPK1 using Necrostatin 1, ameliorated the metabolic syndromes associated with HFD such as glucose intolerance and IR, while having no effect on body weight gain [79] (Table 2).

The last piece of evidence suggesting that necroptosis is implicated in the inflammatory consequences of obesity comes from studies using mice deficient for MLKL. Two independent reports showed that loss of MLKL prevents inflammation, metabolic dysfunction and hepatic steatosis upon HFD [79, 80]. However, neither of these reports link this phenotype with necroptosis. Instead, it appears that MLKL has a direct role in hepatic insulin signalling independently of inflammation [79]. Noteworthy, one of these studies found that MLKL-deficiency prevented body weight gain indicating a potential contribution of MLKL to energy metabolism. Unfortunately, there is little focus on the inflammatory or cell death features in WAT and hence the understanding of the role of MLKL in obesity-induced inflammation requires further investigation.

Apoptosis and necroptosis are highly interlinked cell death pathways. Therefore, in order to understand what is happening at the physiological level requires the study of both cell death modalities in parallel. Importantly, because of the crosstalk between inflammation and energy homeostasis, it is quite crucial to dissect the cell death versus the non-cell death functions of these components in order to clearly understand the processes that regulate obesity-induced inflammation.

## The many faeces of inflammasome activation during obesity

IL-1β is, together with TNF, amongst the most important proinflammatory cytokines known to interfere with insulin signalling, which in the case of IL-1β, it is mediated by targeting IRS-1 [81]. Randomised clinical trials have shown that blockade of IL-1β with Anakinra leads to a sustained reduction in systemic inflammation and improvement of Type 2 Diabetes [82, 83]. Obese and Type 2 Diabetes patients were reported to have elevated expression of all classical inflammasome components, including NLRP3, ASC, Caspase-1 and IL-1β in both liver and visceral adipose tissue. Furthermore, NLRP3 silencing ameliorates LPS-induced inflammation and fibrosis in human visceral adipocytes [84]. Yet, the exact molecular pathway and the cellular systems involved in inflammasome activation and pyroptosis in the context of obesity are not very clear (Table 2).

The role of Caspase-1 during obesity is controversial since some studies reported that its loss results in an increased body weight gain but without metabolic abnormalities [85, 86]. Further insight was provided in a study showing that Caspase-1/11-deficient mice, which have increased body weight gain and liver steatosis upon HFD, as previously reported, present a dysregulation of gut microbiota that modulates liver lipid content leading to steatosis [87]. Interestingly, gut microbiota was also affected in mice lacking NLRP3 in response to HFD. Yet, in this case, NLRP3-null mice were protected from liver steatosis and cardiovascular disease [88]. Whether this is a cell death-mediated phenotype was not explored in any of these reports. However, it is likely that in the absence of Caspase-1/11 or NLRP3, pyroptosis is impaired upon HFD thereby rewiring cell death and inflammatory processes which could greatly affect the microbiome balance in the gut (Table 2).

A recent study reports that both Caspase-1 and NLRP3 are implicated in adipogenesis and insulin sensitivity and, contrary to previous reports, Caspase-1-deficient mice are protected against body weight gain as well as from becoming IR upon HFD [89]. Similar observations were made using a Caspase-1 inhibitor [90]. These findings correlate with a similar protection observed in NLRP3- and IL-1β-deficient mice [89]. Additional studies link inflammasome activation with thermogenesis since IL-1β treatment impaired cAMP-induced elevation of UCP1 and other substrates in mouse and human adipocytes [91]. The authors claim that activation of inflammasome in ATMs during obesity induces the release of IL-1β which interferes with the metabolic capacity of bystander adipocytes [91]. Given the importance of inflammasome activation during obesity due to FFA or production of oxygen species, it has been proposed that obese patients are more susceptible to pathogen infection, mainly by viruses, by mechanisms that depend on pyroptosis of macrophages [92]. This link was particularly prominent in obese patients infected with COVID-19 [92] and NLRP3 inhibitors may be beneficial to these patients.

Contrary to NLRP3 and ASC, which are upregulated in WAT in obese individuals. Other NLR family members including NLRP1, NLRC4, and AIM2 showed similar levels of expression in WAT between lean and obese individuals [93]. In mice, NLRP1 deletion leads to intrinsic lipid accumulation, spontaneous obesity and metabolic syndrome. This phenotype was attributed to IL-18 production since IL-18 is increased in WAT of obese NLPR1-null mice and IL-18-deficient mice also develop metabolic syndrome [94–96]. Furthermore, NLRP1 activation improves glucose tolerance and insulin sensitivity through IL-18-mediated lipolysis [94–96]. Yet, the relevance of these findings in humans remains to be evaluated.

In the last years, inflammasome research has slightly shifted towards understanding the role of Gasdermins, the final executor of pyroptosis, in different pathological conditions [44, 97]. A very recent study showed that patients with NASH and NAFLD have a massive increase in the N-terminal fragment of Gasdermin D, which is the activated form of this killer protein [98] (Fig. 2). In addition, they showed that Gasdermin D loss results in protection from steatohepatitis upon methionine-choline deficient diet or HFD [98]. Curiously, Gasdermin D is apparently involved in lipogenesis in hepatocytes possibly via NF-κB-mediated AMPK regulation as described in previous sections. Whether this is a consequence of pyroptotic cell death or a direct role of Gasdermin D in metabolic processes is not yet clear (Table 2).

In sum, although the implication of inflammasome activation in adipocytes has not been entirely demonstrated, there is no question that macrophage-inflammasome activation triggers systemic inflammation during obesity and it is one of the main culprits of metabolic syndromes. Despite a huge amount of work on the role of inflammasome activation in metabolic disorders, we are still lacking an understanding of pyroptotic cell death in WAT upon obesity and its metabolic implications. Also, the importance of pyroptosis in different cellular compartments can have distinct contributions to inflammation and energy homeostasis and this topic warrants future investigation.

## Concluding remarks

Obesity is a disease that has a multifactorial origin and is associated with multiple metabolic syndromes. Understanding the aetiology of obesity-induced inflammation has, therefore, caught the attention of many scientists and clinicians alike. After a huge amount of work during decades of research, we now understand how the inflammatory signalling cascades that are a hallmark of obesity are highly interlinked with energy homeostasis and insulin signalling. The role of cell death in metabolic syndromes has also received some attention in the last decade given the importance of cell death in regulating inflammation and metabolism. Yet, there is still a long way to go to address important questions, regarding the interplay between inflammation and obesity as well as the crosstalk between different metabolic tissues. At present, what type of cell death prevails during obesity is ill-defined. We also lack a clear picture of whether and how different cell death types that can be triggered by different factors (e.g. cytokines, lipids, DAMPs, PAMPs, etc) affect metabolic inflammation.

In the modern world, the environmental pressure is no longer determined by the lack of nutrients, but rather by their excessive availability. Human beings have not yet adapted to these modern circumstances and hence obesity is a factual clinical issue. Therefore, understanding and defining molecular processes taking into consideration the function of each individual organ is required in order to tackle, either by pharmacological or behavioural means, metabolic syndromes.

## Supplementary information

Glossary
