# Supplementary material for: Cell death and inflammation during obesity: “Know my methods, WAT(son)”
Source: Cell Death Differ. 2022 Sep 29;30(2):279–92. doi: 10.1038/s41418-022-01062-4 (PMC9520110; doi:10.1038/s41418-022-01062-4)
Supplement: Supplementary file 1 — Glossary [file 41418_2022_1062_MOESM1_ESM.pdf]

Glossary

ASC: adaptor molecule apoptosis-associated speck-like protein containing a CARD recruits pro-caspase-1 to the inflammasome.

BMI (Body Mass Index): It is defined as a person's weight in kilograms divided by the square of the person's height in metres (kg/m2). It is a measure of obesity.

Hepatic steatosis: intrahepatic lipid accumulation.

Inflammasome: multi-protein complex form that promotes the inflammatory form of cell death characterise by the pores formation in the plasma membrane named pyroptosis.

***db/db* mice:** genetic model for spontaneous type 2 diabetes and obesity. Homozygous mice carrying a mutation in leptin receptor ($Lepr^{db}$) demonstrate morbid obesity, chronic hyperglycemia, pancreatic β-cell atrophy and become hypoinsulinemic between 4-8 weeks of age.

FAT-ATTAC mice: This transgenic mouse model carries an adipocyte-specific Caspase-8-FKBP fusion protein that allows controllable Caspase-8 dimerisation and activation. Targeted Caspase-8 activation allows the selective ablation of adipocytes by apoptosis upon treatment with a chemical dimeriser.

Insulin resistance: impaired biologic response to insulin stimulation by target tissues, primarily the liver, muscle, and adipose tissue. Diminish glucose disposal results in a compensatory increase insulin production by the β-cells, leading to hyperinsulinemia. Metabolic consequences of insulin resistance can result in hyperglycemia, hypertension, dyslipidemia, visceral adiposity, hyperuricemia, elevated inflammatory markers, endothelial dysfunction and a prothrombic state.

IRS-1: Insulin receptor substrate (IRS) mediates metabolic actions of insulin.
IRS-1 is abundantly expressed in the liver and while IRS-2 expression is suppressed by insulin at the transcriptional level, IRS-1 expression remains intact and is not downregulated by insulin.

Metabolic syndrome: accumulation of several disorders, which together raise the risk of developing atherosclerotic cardiovascular disease, insulin resistance, diabetes mellitus and vascular and neurological complications such as a cerebrovascular accident. Metabolic syndrome is diagnosed when the patience fulfils 3 out of 5 of the following symptoms: exceeded waist circumference, elevated blood triglycerides, reduced high-density cholesterol, elevated fasting glucose and high blood pressure.

Mitochondrial cell death: Cell death pathway characterised by mitochondria outer membrane permeabilization (MOMP); the release of various mitochondrial intermembrane space proteins to the cytoplasm activates Caspase-3/7, resulting in apoptosis. MOMP is often considered a point of no return because it typically leads to cell death, even in the absence of caspase activity.

NAFLD: Nonalcoholic fatty liver disease is a spectrum of hepatic diseases associated with metabolic and cardiovascular disorders, such as obesity, insulin resistance, hypertension, dyslipidemia and type 2 diabetes. It is frequently recognized as the hepatic manifestation of the metabolic syndrome. NAFLD could be defined as an increase in liver fat content in the absence of secondary cause of steatosis.

NASH: Nonalcoholic steatohepatitis. Nonalcoholic fatty liver disease is characterised by lobular inflammation and hepatocyte ballooning. The pathophysiology of NASH is complex and multifactorial. The mechanisms involved in the pathogenesis of NASH, include dietary factors, insulin resistance, genetic polymorphisms, lipotoxicity, and altered gut microbiota.

NLRP: Nucleotide-binding oligomerization domain, Leucine rich Repeat and Pyrin domain containing. Multimeric protein complex that recognise conserved protein domains of intracellular pathogens. Its scaffolding function is need it for the formation of the inflammasome that initiates an the inflammatory form of cell death called pyroptosis.

***ob/ob* mice:** Genetic model of obesity caring a mutation that impairs leptin (Lep) production. Homozygous mice for Lep^{ob} show spontaneous obesity, hyperphagia, transient hyperglycemia, glucose intolerance, and elevated plasma insulin. These mice are used to model phases 1 and 2 of type II diabetes and obesity.

PPARɣ: peroxisome proliferator-activated receptor ɣ. PPARγ is considered the master regulator of adipogenesis, and accordingly has been extensively studied in the context of obesity. Target genes of PPARγ are involved in adipocyte differentiation, lipid storage, and glucose metabolism.

***Zucker$^{fa/fa}$* rats:** Genetic obese rat model characterized by hyperlipidemia, hypercholesterolemia, and hyperinsulinemia that develops adipocyte hypertrophy and hyperplasia. Zucker$^{fa/fa}$ model does not develop type II diabetes, but present fasting hyperglycemia and glucose intolerance, making them useful for studies of the pre-diabetic state.
